# Dramatic undercutting of piedmont rivers after the 2008 Wenchuan Ms 8.0 Earthquake

**DOI:** 10.1038/srep37108

**Published:** 2016-11-18

**Authors:** Niannian Fan, Ruihua Nie, Qiang Wang, Xingnian Liu

**Affiliations:** 1 State Key Laboratory of Hydraulics and Mountain River Engineering, College of Water Resource & Hydropower, Sichuan University, Chengdu, Sichuan 610065, China

## Abstract

Changes in river channel erosion or deposition affect the geomorphic evolution, aquatic ecosystems, and river regulation strategies. Fluvial processes are determined by the flow, sediment and boundary conditions, and it has long been expected that increasing sediment supply will induce aggradation. Here, based on thorough field surveys, we show the unexpected undercutting of the piedmont rivers influenced by the 2008 Wenchuan (Ms 8.0) Earthquake. The rivers flow from the Longmen Mountain with significant topographic relief to the flat Chengdu plain. In the upstreams, sediment supply increased because of the landslides triggered by the earthquake, causing deposition in the upstream mountain reaches. However, the downstream plain reaches suffered undercutting instead of deposition, and among those rivers, Shiting River was the most seriously affected, with the largest undercutting depth exceeding 20 m. The reasons for this unexpected undercutting are proposed herein and relate to both natural and anthropogenic causes. In addition, we also demonstrate, at least for certain conditions, such as rivers flowing from large-gradient mountain regions to low-gradient plain regions, that upstream sediment pulses may induce aggradation in upstream and degradation in downstream, causing the longitudinal profile to steepen to accommodate the increasing sediment flux.

The devastating Ms 8.0 Wenchuan Earthquake (hereafter referred to as the Wenchuan Earthquake) occurred on May 12, 2008, directly triggered tens of thousands of landslides and rock falls^1^ with a total volume of 2.8 × 10^9^ m^3^ 2. The resulting loose materials also induced subsequent landslides and debris flows in the mountain regions^3^. Observing the fluvial and geomorphological processes after such a strong earthquake is of both practical and academic importance^4^,^5^,^6^.

All of the rivers in the Wenchuan Earthquake shock area exhibited relatively steady and equilibrium states before the earthquake. Those rivers flow from the Qinghai-Tibet Plateau to the Sichuan Basin and exhibit very narrow transition zones, in which the rivers flow from the sharp fluctuating mountain regions to the extremely flat plains. The sediment supply increased sharply compared to the pre-earthquake state because of the large amounts of loose materials from the landslides and rock falls in the mountain regions. We conducted more than 40 extensive qualitative field surveys of the rivers in the earthquake shock regions between May 2008 and March 2016, revealing that all of the rivers indeed aggraded to some extent in the mountain reaches, due to large amounts of particles produced by Wenchuan Earthquake excessed the transport capacity by the hydrodynamic condition, which was accordance with previous studies^7^,^8^,^9^. However, the rivers in the plain reaches underwent degradation instead of aggradation.

In contrast to the deposition observed in the mountain reaches after the Wenchuan Earthquake^7^,^8^,^9^, the eroding and undercutting of the plain reaches remain poorly documented and understood. Here, we report the dramatic undercutting of the Shiting River in the plain reaches, which underwent the largest magnitude of undercutting. For comparison, we also report the undercutting in the plain reaches of the Jianjiang and Mianyuan Rivers, which have similar physical geography characteristics and also suffered undercutting, although the magnitudes were significantly smaller. The study area and relevant rivers are presented in Fig. 1.

The Shiting River originates from the Longmen Mountain and flows to the Chengdu Plain. The climate type is subtropical humid monsoon, with an annual average precipitation of 850–1,700 mm that decreases from the upstream mountain area to the downstream plain area. Gaojingguan is the transition zone between the Longmen Mountain and the Chengdu Plain (the mountain exit point), located a hydrological station, which monitors a basin area of 701 km^2^ and a length of 52.7 km. The measured average flow is 20.1 m^3^/s, and floods with flows exceeding a hundred times the annual average flow can be induced by the summer and autumn monsoons. The floods with 2-year, 10-year and 50-year recurrences are 600 m^3^/s, 2,095 m^3^/s and 3,437 m^3^/s, respectively. The average slopes in the mountain and plain reaches are 44‰ and 3.6‰, respectively. All of the reaches in the trunk channel of the Shiting River are mantled by thick alluvial deposits, even after deep undercutting. From Gangjingguan to the mouth, the median sizes of the surface sediment generally decrease (from 50 mm to 15 mm).

The Shiting River converges with the Jianjiang and Mianyuan Rivers successively to form the Tuojiang River, which is a first-order tributary of the Yangze River. The Shiting, Mianyuan and Jianjiang Rivers have similar physical geography characteristics. In particular, all three rivers flow from mountainous regions with great topographic relief to extremely flat plain regions, with 2.7, 2.0 and 4.2 × 10^8^ m^3^ 2 loose materials triggered by Wenchuan Earthquake in the upstream mountain regions, respectively. No controlled reservoirs have been built in these three rivers.

The undercutting depths were measured by the traces in the artificial structures (for e.g., the elevation difference from the middle of pier foundation cap to the river bed) across the rivers (Fig. 2 and Methods). Note that some of the measured points had no sills (Fig. 2b), whereas others had sills to prevent further undercutting (Fig. 2a). These sills could protect the reaches upstream, but at the toe of each sill, scarp undercutting appeared due to local sour. In these cases, we measured each undercutting depth both above and below the sill’s crest. Uncertainties of the undercutting measurements were estimated to be 0.5 m. It is difficult to evaluate the exact effect of the sills quantitatively; however, the bed would suffer more degradation without the sills^10^.

Figure 3 shows the dramatic undercutting of the Shiting River; the inset plot presents the profile for the entire Shiting River, and the main plot shows the profiles for the plain reaches (from Gaojingguan to the mouth). The original profile is shown by the blue line, and the profile before the 2015 flood season (7 years after the Wenchuan Earthquake) is indicated by the red line. The maximum measured undercutting depth was 20.8 m at the 105^th^ Provincial Highway Bridge. In general, the undercutting depth increased from Gaojingguan to the 105^th^ Provincial Highway Bridge and then decreased to the mouth (see Extended Data for details). The undercutting volume was calculated to be 1.0 × 10^8^ m^3^ (see Methods), by comparison, the aforementioned loose material triggered by the Wenchuan Earthquake in the mountain regions of Shiting River was 2.7 × 10^8^ m^3^ 2.

The undercutting depth decreased to zero in the reaches close to the mouth. We interpret that a 15-km sandstone bedrock exposure reach less than 2 km downstream of the mouth may serve as the base level to prevent the river from undercutting (Fig. 1). The abrasion of the bedrock was negligible compared to the undercutting of the alluvial reaches from the Wenchuan Earthquake to the present.

For Jianjiang and Mianyuan Rivers, undercutting also occurred in the plain reaches, but the magnitudes were much smaller than those observed in the Shiting River. The largest undercutting depths along the Jianjiang and Mianyuan Rivers were 4.5 m in the Renmin Channel Culvert and 5.8 m in the 105^th^ Provincial Highway Bridge, respectively, and undercutting depths exceeding 4 m were fairly localized. The undercutting volumes for the Mianyuan and Jianjiang Rivers were 8.7 × 10^6^ m^3^ and 6.2 × 10^6^ m^3^, respectively, at least one order smaller than those for the Shiting River (1.0 × 10^8^ m^3^). We also need to state that the average uplift caused by Wenchuan Earthquake in the mountainous regions was only up to 3 cm^11^, negligible to the undercutting of our studied rivers’ beds.

The undercutting magnitudes vary greatly between years. After the earthquake, serious floods occurred in Shiting River in 2008, 2010 and 2013 with peak discharges of 1730, 1030 and 2310 m^3^/s, respectively. Most of the undercutting occurred in these three years, as did most of the deposition in the mountain reaches. Thus, the mountain reaches are “flood-depositing streams,” whereas the plain reaches are “flood-cleaning streams”^12^.

In summary, the piedmont rivers in the earthquake shock regions suffered degradation, which challenged the common notion that sediment pulses induce aggradation. Below, we propose several possible explanations from both anthropogenic and natural perspectives.

Commercial sediment extraction is one anthropogenic explanation. After the Wenchuan Earthquake, reconstruction required a large amount of materials, and thus, sediment extraction from the rivers exceeded that before the earthquake, possibly disrupting the equilibrium state, particularly because of the destruction of the armoring layer^13^.

From the natural perspective, we propose the following two explanations:

1. Fine particles mobilize coarse particles^14^,^15^,^16^. In the mountain regions, the earthquake triggered loose materials, and the size of the particles varies greatly from clay to boulders. Because of sorting processes^17^,^18^, most of the large particles remained in the upstream reaches for the critical incipient shear stress for the large particles excess the actual shear stress, whereas the small particles were transported downstream, mobilizing the original particles in the riverbed^14^,^15^,^16^ and resulting in degradation. The mountain exit point acts as a hinge, in which reaches upstream of mountain exit point aggrade and downstream of mountain exit point degrade. The hinge phenomena also results from the channel width control. Upstream of mountain exit point, stream is narrow so that the fine particles could fully cover the riverbed. However, downstream of mountain exit point, stream is wide so that fine particles only partially cover the riverbed, and thus the previous coarse particles are more mobile.
2. The critical shear stress for incipient motion increases with slope^19^,^20^. The average slope of the plain reaches of the Shiting River is 3.6‰, whereas that of the mountain reaches is more than 10 times higher. As a result, the particles in the steeper reaches may need higher critical shear stress to entrain than those in gentler reaches of our studied rivers.

The more serious undercutting observed in the Shiting River than other rivers require further analysis. We first excluded commercial sediment extraction as a primary reason. Although the exact volume of extraction was unavailable because part of the sediment-extraction activities were performed without a license, during our field surveys, we observed that all rivers were subjected to sediment extraction and found no obvious indication suggesting that more sediment was extracted from Shiting River than from other rivers.

Why did Shiting River suffer such serious undercutting? Our field survey indicated that the phosphate mines in the upstream mountain regions of the Shiting River played important roles both upstream and downstream. The phosphate mines arose decades ago and remained in use after the earthquake. The mining activities further loosened the materials triggered by the earthquake, increasing the downstream transport of fine particles and, thus, mobilizing the particles in the downstream riverbed. Although the exact volumes of transported particles of different sizes are unknown, our visual interpretation during the field survey suggested that in the non-flood seasons, the Shiting River’s flow in the mountain reaches was extremely muddy, whereas that in other rivers was extremely clean.

In Fig. 4, we present a scheme for river profile adjustment for certain conditions in the rivers studied here. We stress again all the reaches in the trunk channel of the Shiting River as we study are mantled by thick alluvial deposits, even after deep undercutting, so the cover and tool effects for bed rock bedrock substrate-type rivers^21^,^22^ are not applicable here. The proposed scheme is as below: Rivers flow from mountain regions with great topology relief to plain regions. Non-uniform sediment pulses in the mountain regions upstream may result in aggradation in the upper reaches and degradation in the lower reaches, and the mountain exit point acts as a hinge where the longitudinal profile grows steeper to transport the increasing sediment flux.

We acknowledge that the unexpected undercutting that occurs after a large sediment pulse remains incompletely understood, and we suggest two avenues for future studies. First, persistent and detailed observations of rivers shocked by the Wenchuan Earthquake are still needed. Second, flume experiments and numerical simulation studies of river adjustments are necessary to address non-uniform sediment pulses with complex boundary conditions.

## Methods

### Stream net extraction

**Figure 1:**
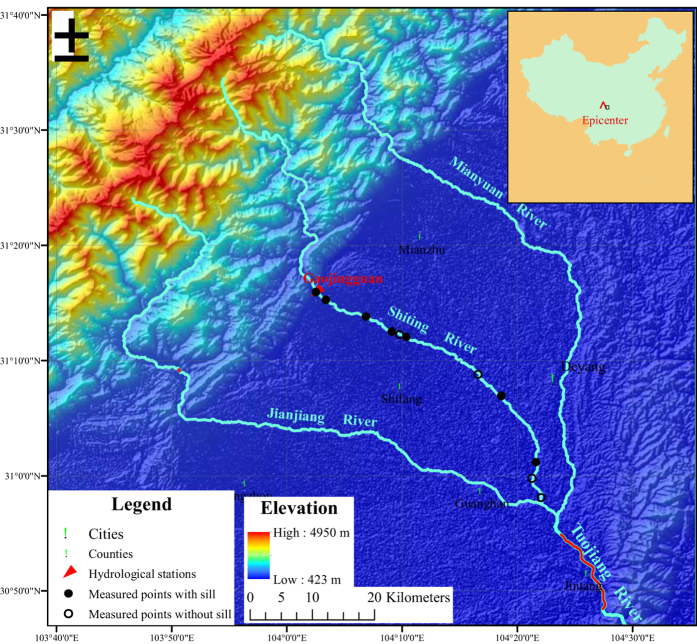
Sketch map of the studied rivers. The inset map (top right) shows the relative location of the study area in China and the epicenter of the 2008 “5.12” Wenchuan Earthquake (Ms 8.0). The Shiting, Jianjiang and Mianyuan Rivers all flow from the Longmen Mountain with great topology relief to the extremely flat Chengdu Plain, and the three rivers converge to form the Tuojiang River. The bedrock exposure reaches are marked in red and otherwise are covered by fluvial deposits. The measured points with or without sills along the Shiting River are marked as solid and hollow circles, respectively. The map was created by Arcgis 10.2 (http://www.esri.com/software/arcgis/arcgis-for-desktop).

We extracted the stream net using the ArcGIS software from Digital Elevation Model (DEM) data, which were downloaded from http://srtm.csi.cgiar.org/SELECTION/inputCoord.asp. The DEM data were collected in February 2000 with a resolution of 90 m × 90 m. We filled the DEM and extracted the stream based on the direction of the filled DEM with a conflux threshold of 2,000 pixels. The extracted stream net fitted the actual rivers well, as shown in Fig. 1. The river length, basin area and longitudinal profiles, as shown in Fig. 3, were based on our extracted stream net.

### Measuring the undercutting depth and calculating the undercutting volume

**Figure 2:**
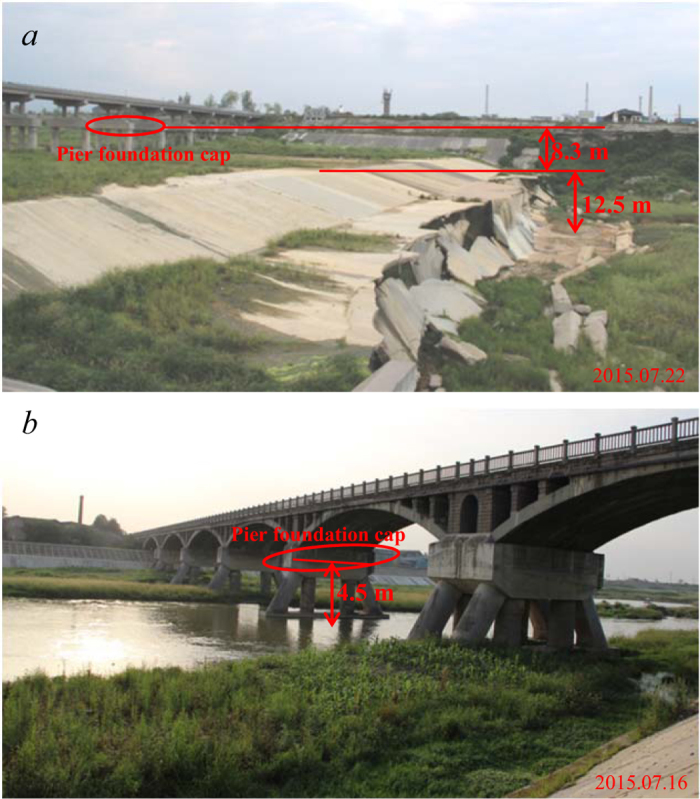
The undercutting depth measured by the traces in the artificial structures (for e.g., the elevation difference from the middle of pier foundation cap to the river bed). Several of the measured points were built with sills (**a**, the 105^th^ Provincial Highway Bridge across the Shiting River) to prevent further undercutting, whereas others had no sill (**b**, the Jinyu Bridge across the Shiting River, note that the inclined piers were built after the river undercutting to protect the bridge, while the vertical piers were built at the same time with the bridge). For the former cases, we measured the undercutting depth both above and below the sill crest.

Because of the lack of large-scale topographical maps covering different periods, we measured the undercutting depth from the traces in the artificial structures, which were built before the Wenchuan Earthquake. The pier foundation caps for bridges were originally partly buried but became suspended after undercutting (we also verified the riverbed level before the earthquake with local residents). We measured the elevation difference between the center of the pier foundation caps to the riverbed, as shown in Fig. 2b (the Jinyu Bridge across the Shiting River) using a laser range finder. Several of the measured points were built with the sills as shown in Fig. 2a (the 105^th^ Provincial Highway Bridge across the Shiting River) to prevent further undercutting, and in these instances, we measured the undercutting depths both above and below the sill crests. Uncertainties of the undercutting measurements were estimated to be 0.5 m.

**Figure 3:**
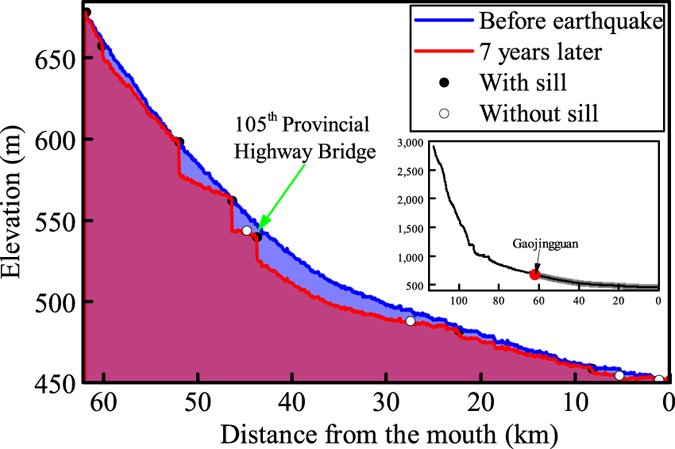
The longitudinal undercutting of the Shiting River in the plain reaches. The inset plot shows the longitudinal profile for the entire Shiting River, and the main plot presents the enlarged plot of the longitudinal profile of the plain reaches (downstream of Gaojingguan). The blue line is the original profile before the earthquake, and the red line is the profile 7 years after the earthquake (July 2015, before the flood season).

We measured 11 points along the Shiting River in 2015 before the flood season, and the longitudes and latitudes of the measured points were recorded. Based on the original longitudinal profile of the Shiting River, we determined the distances to the mouth from the measured points and plotted the profiles after undercutting. Note that according to the measured points around the sills, the river channel was undercut gradually upstream but exhibited scarp undercutting at the toes (Fig. 2a), as shown in Fig. 3. The undercutting depths between the measured points were interpolated linearly.

The undercutting volume was calculated as


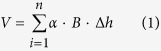


where *n* is the number of subsections (here, 733); *α* is the form coefficient of the river cross-section, which was set to be 2/3; *B* is the channel width obtained from satellite images, which ranged from 240 m to 360 m; and Δ*h* is the undercutting depth obtained above. The calculated undercutting volume for Shiting River was 1.0 × 10^8^ m^3^.

### Distinguishing alluvial and bedrock exposure channels

We distinguished alluvial and bedrock exposure channels (Fig. 1) primarily by direct visual interpretion during the non-flood season. However, part of the reaches may be difficult to approach, and thus, we used an unmanned aerial vehicle equipped with a camera from DJI company (http://www.dji.com/cn/company) to take photos. These photos, which were used to interpret the river channel type, had a resolution of 4000 × 3000, and the pixel length was less than 20 cm. We also compared the results obtained based on direct visual interpretation and photos to avoid misinterpretations.

## Additional Information

**How to cite this article**: Fan, N. *et al*. Dramatic undercutting of piedmont rivers after the 2008 Wenchuan Ms 8.0 Earthquake. *Sci. Rep.* **6**, 37108; doi: 10.1038/srep37108 (2016).

**Publisher’s note**: Springer Nature remains neutral with regard to jurisdictional claims in published maps and institutional affiliations.

## Supplementary Material

Supplementary Information

## Figures and Tables

**Figure 4:**
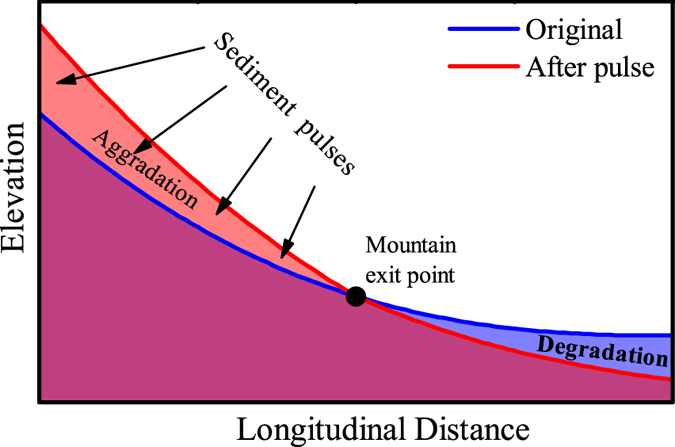
Scheme diagram of the longitudinal profile adjustment of rivers flowing from mountains to plains with sediment pulses in the mountain area. The mountain exit point acts as a hinge, and the mountain reaches aggrade as the plain reaches degrade. As a result, the slope increases to deliver more sediment flux.
