# Supplementary material for: Dramatic undercutting of piedmont rivers after the 2008 Wenchuan Ms 8.0 Earthquake
Source: Sci Rep. 2016 Nov 18;6:37108. doi: 10.1038/srep37108 (PMC5114544; doi:10.1038/srep37108)
Supplement: Supplementary Information [file srep37108-s1.doc]

Supplementary Information

**Dramatic undercutting of piedmont rivers after the 2008 Wenchuan Ms 8.0 Earthquake**

Niannian Fan, Ruihua Nie^*^, Qiang Wang, and Xingnian Liu

State Key Laboratory of Hydraulics and Mountain River Engineering, College of Water Resource & Hydropower, Sichuan University, Chengdu, Sichuan 610065, China.

^*^Correspondence to Ruihua Nie (scunie@163.com)

**Table 1**. **Measured undercutting of Shiting River.**

| Location | Date of measurement | Longitude | Latitude | Distance From the month | Undercutting depth (m) | Sill |
| --- | --- | --- | --- | --- | --- | --- |
| Gaojingguan | 2015.7.22 | 104.0396 | 31.2766 | 62.00 | 0+0 | Yes |
| Guangluo Bridge | 2015.7.22 | 104.0562 | 31.2544 | 60.13 | 2.8+7.1 | Yes |
| Hongyan Branch Channel | 2015.7.22 | 104.1109 | 31.2304 | 51.94 | 0+19.50 | Yes |
| Renmin Channel | 2015.7.22 | 104.1493 | 31.2108 | 46.35 | 0+18.80 | Yes |
| 2^rd^ Cheng-Mian Super Highway Bridge | 2015.7.22 | 104.1608 | 31.2072 | 44.86 | 10.10 | No |
| 105^th^ Province Highway Bridge | 2015.7.22 | 104.1729 | 31.2006 | 43.72 | 8.3+12.5 | Yes |
| 106^th^ Province Highway Bridge | 2015.7.22 | 104.2766 | 31.1464 | 27.50 | 6.90 | No |
| 108^th^ National Highway Bridge | 2015.7.23 | 104.3474 | 31.0738 | 22.37 | 1.9+3.1 | Yes |
| Cheng-Mian Super Highway Bridge | 2015.7.23 | 104.3610 | 31.0205 | 8.27 | 1.1+1.9 | Yes |
| Jinyu Bridge | 2015.7.16 | 104.3558 | 30.9965 | 5.36 | 4.50 | No |
| Shuangjiang Bridge | 2015.7.16 | 104.3677 | 30.9685 | 1.17 | 0 | No |

For measured locations with sills, undercutting depths are recorded as “*a*+*b*”, in which “*a*” and “*b*” are the undercutting depths above and under the sills’ crests respectively, see methods for details.
